# Performer-KAN-Based Failure Prediction for IGBT with BO-CEEMDAN

**DOI:** 10.3390/mi16060689

**Published:** 2025-06-08

**Authors:** Yue Xiao, Fanrong Wang

**Affiliations:** School of Electrical and Electronic Engineering, Hubei University of Technology, Wuhan 430070, China; xy06232025@163.com

**Keywords:** IGBT, PHM, failure prediction, Performer, KAN

## Abstract

Insulated Gate Bipolar Transistors (IGBTs) are widely deployed in power electronic systems due to their superior performance. However, at the same time, they are one of the most critical and fragile components in electronic systems. The failure prediction of IGBTs can precisely forecast the potential risk to guarantee system reliability. In this paper, Bayesian-optimized CEEMDAN is adopted to extract fault features efficiently, and a prognostic model named Performer-KAN is proposed for IGBT failure prediction. The proposed model combines the efficient FAVOR+ mechanism from the Performer with the flexible spline-based activation of the Kolmogorov–Arnold Network (KAN), enabling improved nonlinear approximation and predictive precision. Comprehensive experiments were conducted using the IMFS, which were decomposed by BO-CEEMDAN. The model’s performance was evaluated using key metrics such as MAE, RMSE, and R^2^. The Performer-KAN demonstrates superior prediction accuracy while maintaining low computational overhead, compared to six representative deep learning models. The results demonstrate that the proposed method offers a practical and effective solution for real-time IGBT health monitoring and fault prediction in industrial applications.

## 1. Introduction

Power electronic systems are fundamental components in energy conversion and control, where the reliability of power devices is critical to the overall system performance. Among them, the Insulated Gate Bipolar Transistor (IGBT) has become one of the most widely used power semiconductor devices due to its high input impedance and low on-state voltage drop [1]. In smart grids, IGBTs act as core switching elements in inverter topologies, motor drive systems, and high-voltage direct current (HVDC) transmission systems for energy regulation [2,3]. In new energy vehicle powertrains, IGBTs are integrated into the motor control unit to realize power conversion functionalities [4]. In renewable energy applications, IGBTs significantly enhance the energy conversion efficiency of wind power converters and photovoltaic inverters by optimizing switching loss characteristics [5].

However, due to continuous operation under extreme conditions such as high temperature, high voltage, and high current, the IGBTs’ reliability issues have become increasingly prominent, posing a critical challenge to extending the system lifespan and improving the overall performance. According to relevant statistical data, IGBT failures are among the leading causes of power electronic system malfunctions [6,7]. Therefore, conducting reliability assessments and lifetime predictions of IGBTs is of significant engineering importance and application value.

Prognostics and Health Management (PHM) technology is a systematic engineering methodology that combines signal processing and data analysis techniques [8]. Applying PHM techniques to power electronic devices allows for a systematic and efficient assessment of the device reliability. In recent years, PHM-based reliability research on power electronic devices has emerged as a significant research focus. Based on the PHM framework, this paper conducts a failure prediction of IGBTs, explicitly focusing on estimating their remaining useful life (RUL).

Existing studies on IGBT failure prediction can broadly be classified into physics-based and data-driven modeling. The former investigates the theoretical basis of the degradation process through specific mathematical formulations, typically requiring the assumption that dynamic systems can be precisely modeled and requiring extensive expertise in degradation mechanisms [9]. However, the highly nonlinear nature of complex systems and the diversity of failure modes significantly constrain the practical application of physics-based methods. Consequently, driven by advancements in data acquisition and processing technologies, data-driven methods have emerged as the mainstream approach in fault prediction research [10].

Common data-driven fault prediction models include statistical models, including Particle Filtering [11,12], Markov Process [13], Gaussian Process Regression [14], and Kalman Filter [15,16]; machine learning models, such as Support Vector Machine (SVM) [17], Convolutional Neural Network (CNN) [18], Decision Tree, and K-Nearest Neighbors (KNN) [19,20]; deep learning models, including Recurrent Neural Network (RNN) [21], Long Short-Term Memory (LSTM) [22,23,24], and Transformer-based models [9]. Although these methods have achieved notable progress, they still exhibit several limitations. Statistical models typically rely on strong prior assumptions, restricting their performance when dealing with high-dimensional, nonlinear degradation data. Machine learning models heavily depend on manual feature extraction and selection and often struggle to capture complex temporal dependencies.

Recent research on IGBT fault prediction has mainly focused on improving classical deep learning models through structure fusion, optimization algorithms, and uncertainty modeling to enhance the prediction accuracy and robustness. One common approach is model fusion to strengthen the feature representation. For example, refs. [25,26] combined CNN with LSTM or Transformer to leverage CNN’s local feature extraction. However, such combinations often increase the model complexity and risk overfitting due to the growing number of parameters. In [27], a stacked denoising autoencoder (SDAE) was integrated with LSTM to enhance the data quality via denoising, thereby improving the prediction performance. Nevertheless, this method is sensitive to noise-handling parameters and less stable during training. Some works adopted intelligent optimization algorithms—such as dung beetle optimization (DBO) [28], particle swarm optimization (PSO) [29], improved sparrow search algorithm (ISSA) [30], and sailfish optimization (SFO) [31]—to tune hyperparameters and model structures. Although these methods offer performance gains, they tend to rely on prior experience and are prone to local optima, which may affect the stability and generalization. For uncertainty modeling, refs. [9,32] applied Monte Carlo Dropout to LSTM and Transformer, enabling the quantification of output uncertainty. This improves the robustness and interpretability but increases the training and inference costs. Bidirectional temporal models like Bi-GRU [33] and BiLSTM [34] capture both forward and backward dependencies, slightly improving the prediction accuracy. However, their enhancements over unidirectional models are often limited and may not justify the increased structural complexity.

While deep learning models have demonstrated remarkable capabilities in prediction, they still face the following challenges:

(a) Feature extraction and data processing: The raw accelerated aging experimental data often contain substantial redundant information. Directly feeding such data into prediction models can hinder effective fault feature extraction, leading to reduced prediction accuracy and increased computational burden. Therefore, the appropriate data preprocessing and feature extraction methods are required to provide high-quality inputs for subsequent fault prediction tasks.

(b) Model robustness and generalization: Deep learning models are susceptible to data distribution shifts under complex operating conditions, resulting in potential overfitting or underfitting in fault prediction. Conventional neural networks typically employ fixed activation functions (e.g., ReLU, SiLu), which lack the adaptability to varying data distributions, particularly during the early stages of device aging or under rapidly changing operating conditions, leading to a decline in the prediction accuracy.

(c) Balancing prediction accuracy and computational efficiency: Transformer models exhibit superior performance in capturing global dependencies over long time series through self-attention mechanisms. However, their computational complexity scales quadratically with the sequence length (O(*n*^2^)). Furthermore, achieving an optimal performance often requires stacking multiple layers with hundreds of millions of parameters, imposing significant demands on the computational resources and training data. These challenges limit the direct application of standard Transformer models to large-scale IGBT fault prediction tasks. Thus, reducing the computational complexity while maintaining prediction accuracy remains an open problem.

**Figure 1:**
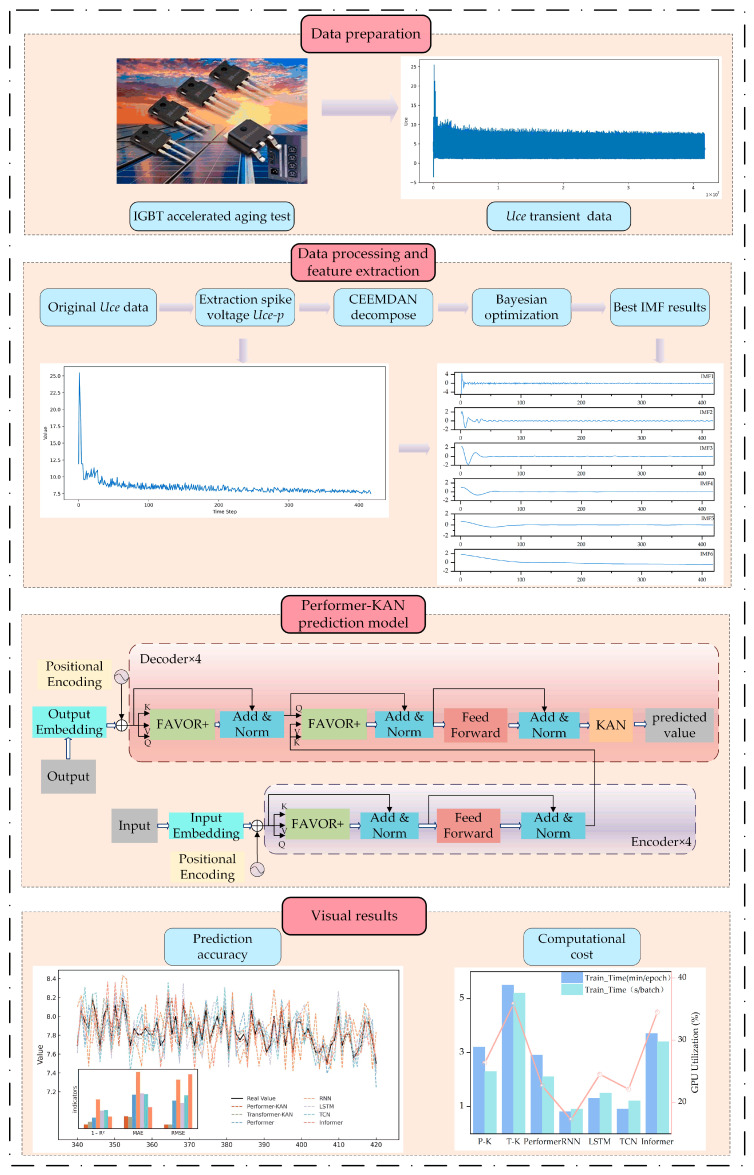
Prediction steps based on the Performer-KAN model.

Inspired by the challenges above, this paper proposes a Performer-based IGBT fault prediction method that, for the first time, combines the Performer architecture with the Kolmogorov–Arnold Network (KAN) and applies it to IGBT fault prediction tasks. The overall technical procedure is illustrated in Figure 1.

To solve the limitation (a), the adaptive noise complete ensemble empirical mode decomposition (CEEMDAN) technique is introduced to decompose the original accelerated aging experimental data into multiple Intrinsic Mode Functions (IMFs) across different frequency bands, thereby enabling the efficient extraction of key fault features and providing high-quality data support for subsequent model construction. To solve the limitation (b), the KAN is incorporated as the fundamental network module, employing Spline Activation Functions to replace the fixed activation functions commonly used in traditional neural networks. This design improves the nonlinear approximation capacity while reducing the model complexity, reducing the risk of overfitting, improving the generalization ability, and enhancing the adaptability to complex degradation patterns. To solve the limitation (c), the proposed method adopts the FAVOR+ (Fast Attention Via positive Orthogonal Random features) mechanism within the Performer framework, which utilizes orthogonal random feature mapping to approximate Softmax attention. This approach reduces the computational complexity of the attention mechanism from O(*n*^2^) to O(*n*), significantly improving the computational efficiency and reducing the memory consumption, making it more suitable for large-scale IGBT fault prediction applications.

The main highlights of the proposed method are summarized as follows:

(1) The original signal is decomposed using the CEEMDAN technique, and Bayesian optimization is employed to adjust the key parameters, thereby automatically minimizing the signal reconstruction error. The best IMF sequences obtained will provide higher-quality input features for subsequent modeling.

(2) First attempt to design a Performer-based prognostic framework with the KAN to enhance the model’s generalization capability through trainable spline-based activation functions, enabling effective adaptation to varying data distributions under different operating conditions.

(3) The FAVOR+ mechanism is introduced to reduce the attention module’s computational complexity from O(*n*^2^) to O(*n*) while maintaining high prediction accuracy, thereby improving computational performance and scalability for large-scale IGBT fault prediction tasks.

## 2. Selection of IGBT Failure Precursor Parameters and Feature Extraction

### 2.1. IGBT Failure Parameter Selection

Common IGBT fault parameters include collector current (*I_C_*), gate-emitter threshold voltage (*U_ge,th_*), collector-emitter voltage (*U*_ce_), and turn-on/turn-off time (*t*_on_*/t*_off_) [35]. This paper systematically analyzes and identifies the most suitable precursor parameters for failure prediction, based on failure mechanisms and accelerated aging experimental data.

Abnormal *I_C_* increases may result from load short circuits, drive signal faults, or parasitic conduction. Latch-up conduction of internal parasitic thyristors can also trigger sudden *I_C_* surges. The *I_C_* responds rapidly to transient faults such as short circuits and overcurrents but is susceptible to disturbances from load fluctuations; under normal conditions, the *I_C_* typically varies with load changes.

The gate-emitter threshold voltage (*U*_ge,th_) reflects degradation phenomena such as gate oxide deterioration and gate drive circuit failures and may exhibit drift, reduction, or abnormal fluctuations. Nevertheless, the *U*_ge,th_ is sensitive to temperature and voltage stresses, and environmental variations can destabilize measurements, affecting the fault detection reliability. Variations in drive resistance or capacitance parameters can also prolong *t*_on_*/t*_off_. During normal aging processes, the *U*_ce_ gradually increases, due to rising internal parasitic resistance, a shortened carrier lifetime, and increased package thermal resistance. In cases of packaging failure, an increase in the Equivalent Series Resistance (ESR) directly elevates the steady-state value of the *U*_ce_. In contrast, chip failures (e.g., gate oxide breakdown) induce transient *U*_ce_ aberrations through carrier concentration fluctuations. In summary, the *U*_ce_ can comprehensively reflect multiple types of IGBT failure modes.

During the early stage of this study, the parameters mentioned above were considered for failure prediction. However, further analysis and preliminary experiments showed that the *I*_C_ showed poor stability and was highly sensitive to load fluctuations, which introduced noise and reduced its reliability. The measurement of *t*_on_/*t*_off_ was relatively complex and difficult to standardize, making the accuracy of timing-based features difficult to ensure across different experimental conditions. The *U*_ge,th_ displayed a limited sensitivity to degradation and was not effective in signaling early fault trends. In contrast, the *U*_ce_ provided a good balance between sensitivity and measurement feasibility. It was easy to acquire using standard probes, demonstrated stable performance under varying conditions, and involved a low measurement cost. Therefore, this paper selects the *U*_ce_ as the core parameter for IGBT failure prediction.

The natural aging IGBT experiment is usually not a feasible solution because collecting all source materials from such a long failing time is nearly impossible. Accelerated aging tests shorten the experimental duration by artificially increasing the operational stress or workload without altering the failure mechanisms. NASA provides an open-access database containing datasets from accelerated aging experiments, recording several IGBT characteristic parameters, including transient and steady-state data. Transient data captures rapid dynamic changes during system state transitions, characterized by high-frequency oscillations, focusing on switching processes, dynamic responses, and transient behaviors. In contrast, steady-state data are measured after the system stabilization and reflect long-term operational characteristics, thermal stability, and aging effects.

In the accelerated aging experiment, the tested IGBT was an IRF-G4BC30KD device(Infineon Technologies AG, Neubiberg, Germany) with a TO-220 package, a rated voltage of 600 V, and a rated current of 15 A. To enhance the thermal stress and accelerate the degradation process, no heat sink was applied during the experiment. A PWM gate drive signal with a frequency of 10 kHz and a duty cycle of 40% was continuously applied to the IGBT. Accelerated aging was achieved through thermal cycling, where the gate signal was disabled when the device temperature exceeded 330 °C and re-enabled when the temperature dropped below 329 °C, forming an automatic feedback loop. The protection temperature was set to 345 °C. The IGBT failed after 418 complete switching cycles, with 100,000 high-resolution transient data points collected per cycle for subsequent degradation analysis and feature extraction.

However, IGBTs undergo frequent turn-on and turn-off operations as power devices, where transient voltages, current spikes, and rapid junction temperature variations often trigger failures. Therefore, this study selects transient data as the research focus and employs it as failure parameters for IGBT lifetime prediction. The transient dataset records 418 switching process parameter groups, each containing 100,000 sampling points.

These 418 groups of switching process data were all obtained from the same IGBT device, which was subjected to continuous thermal-electrical stress during the accelerated aging test. Each group corresponds to a complete PWM-controlled switching cycle, capturing voltage and current behaviors within a single period. As the aging progressed, the sequential cycles reflected the gradual degradation of the same device, providing a continuous dataset that enables the model to learn the entire failure evolution process.

**Figure 2:**
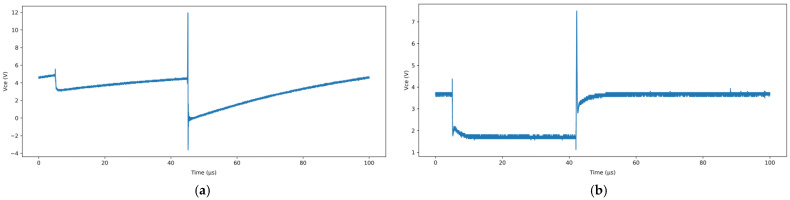
The trend of the *U*_ce_ variation during the switching cycle of the accelerated aging test (**a**) presents the variation of the *U*_ce_ during the first switching cycle, (**b**) presents the variation during the final switching cycle before the device failure.

Figure 2a presents the variation of the *U*_ce_ during the first switching cycle, while Figure 2b presents the variation during the final switching cycle before the device failure. It can be observed that in the early stages of the accelerated aging progression, the *U*_ce_ exhibits relatively stable behavior. In contrast, during the later stages, the fluctuation amplitude of *U*_ce_ increases significantly, revealing clear signs of degradation, which indicates a substantial decline in the IGBT performance.

A large volume of *U*_ce_ data was collected in the accelerated aging experiments, reaching specific sampling points. Direct processing of such high-dimensional data faces two significant challenges: a high proportion of redundant information leads to exponential growth in computation, and signal noise coupled with parameter interactions mask potential fault features, hindering the efficient extraction of sensitive degradation indicators by traditional time- and frequency-domain methods. Therefore, further screening of the raw data is necessary to optimize the fault prediction model’s training efficiency and generalization ability.

**Figure 3:**
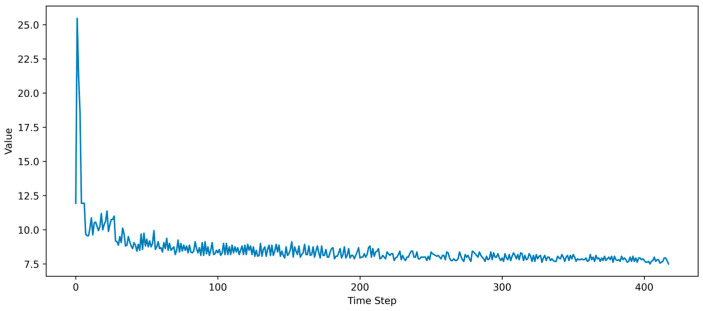
Collector-emitter turn-off transient spike voltage (*U*_ce-p_).

During the continuous turn-on and turn-off cycles under the combined influence of the PWM signals and temperature thresholds, parasitic transistors inhibit the growth of the anode current during the turn-off phase, generating a transient voltage between the collector and emitter, aligned with the supply voltage. This transient is superimposed on the supply voltage, forming a spike voltage higher than the supply voltage, referred to as the collector-emitter transient spike voltage (*U*_ce-p_). The decay waveform of the collector-emitter turn-off transient spike voltage is shown in Figure 3. As the IGBT ages, the *U*_ce-p_ amplitude gradually decays and stabilizes before the complete device failure.

### 2.2. Bo-CEEMDAN-Based Failure Feature Extraction

CEEMDAN decomposes an original time series into subsequences of different frequencies, known as IMFs [36]. The underlying Empirical Mode Decomposition (EMD) method utilizes the data’s intrinsic time-scale characteristics for modal decomposition, making it suitable for non-stationary and nonlinear time series analysis. Building on this, CEEMDAN gradually adds adaptive white noise at each iteration to mitigate noise interference in the decomposition process. CEEMDAN achieves a near-zero reconstruction error with fewer ensemble runs, significantly alleviates modal aliasing, and improves the decomposition and reconstruction accuracy. It also avoids the computational inefficiency of EEMD, which requires many integrations to reduce the reconstruction error.

**Figure 4:**
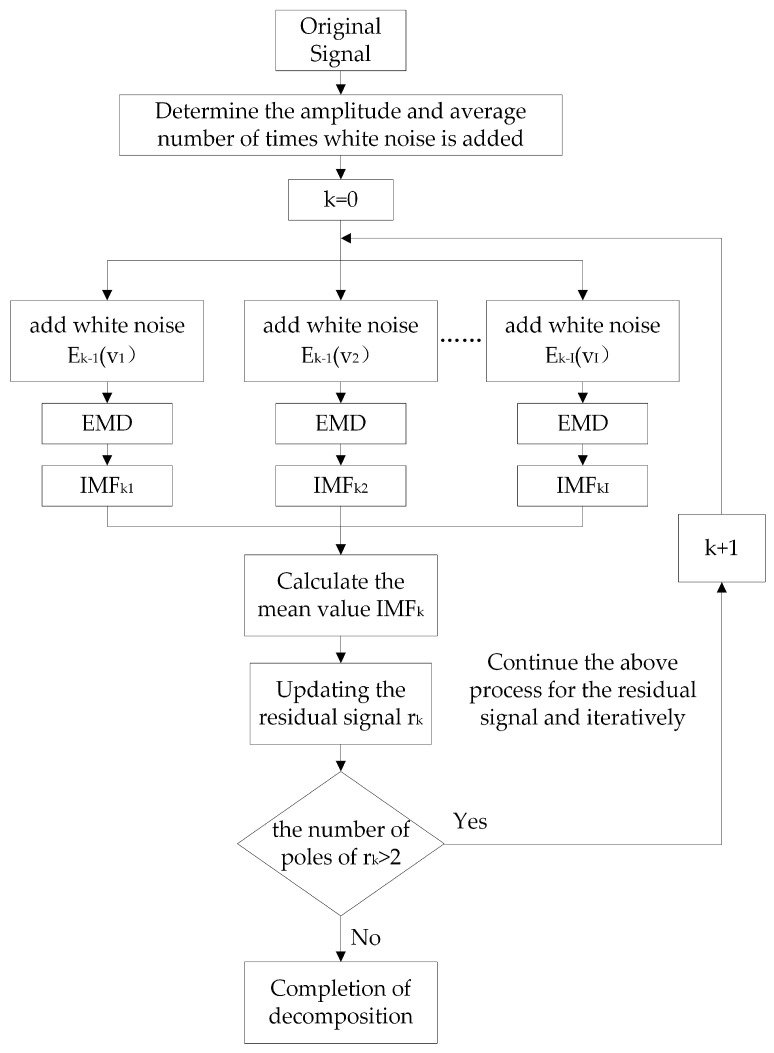
CEEMDAN algorithm flowchart.

The algorithm flowchart of CEEMDAN is illustrated in Figure 4. For a given sequence of original signals *x*(*t*), construct *i* signals after adding noise, where *v_i_*(*t*) is a sequence of white noise with standard normal distribution added in the ith experiment (mean zero, variance 1), is a tuning parameter of the noise amplitude, and *I* denotes the number of experiments. The obtained noise signal is expressed as:

(1) $$x_{i}(t)=x(t)+\varepsilon v_{i}(t)$$

EMD decomposition is employed, and the resulting IMFs are averaged to extract the kth-order IMF component. The extracted IMF is then removed $x_{i}(t)$, and the decomposition process is repeated on the remaining residual signals until the residuals become monotonic functions or approach zero, at which point further decomposition is not possible. The process concludes with the extraction of the complete set of IMF components.

The formula for the IMF and residual signals is given in Equations (2) and (3).

(2) $$IMF_{k}=\frac{\sum_{i=1}^{I}IMF_{ki}}{I}$$

(3) $$r_{k}(t)=x_{k-1}(t)-IMF_{k}$$

The CEEMDAN decomposition involves two key parameters: the noise amplitude and the number of integrations. The former determines the intensity of the added perturbation signal, while the latter controls the number of averaged samples. Since both parameters significantly influence the decomposition quality, this study adopts Bayesian Optimization (BO) based on Gaussian Process Regression (GPR) to identify their optimal combination by minimizing the signal reconstruction error [37]. The objective function is defined as the mean squared error (MSE) between the reconstructed and original signals:

(4) $$MSE=\frac{1}{N}\sum_{i=1}^{N}(s_{\left(i\right)}-{\hat{s}}_{\left(i\right)}{)}^{2}$$

where $s_{\left(i\right)}$ denotes the original signal, ${\hat{s}}_{\left(i\right)}$ denotes the signal obtained from IMF reconstruction, and *N* is the data length. To perform parameter optimization, this paper further defines the parameters to be tuned and their search space, where ξ denotes the noise amplitude and *K* denotes the number of integrations.

(5) $$\left\{\begin{array}{l} \xi \in [0.01,\;0.5]\\ K\in \left\{10,\;11,\;\ldots ,\;50\right\} \end{array}\right.$$

GPR is used to approximate this function, employing a Radial Basis Function (RBF) kernel to construct the covariance matrix and predict the mean and variance for any parameter combination. To balance exploration and exploitation, the acquisition function guides the selection of the following evaluation point. In this work, the Expected Improvement (EI) criterion is adopted, with the current best MSE denoted as $f_{\min}$.

(6) $$\mathrm{EI}(x)=E[\max(f_{\min}-f(x),\;0)]$$

The model is iteratively trained and evaluated based on selected parameter sets. After each update, the objective function values are recalculated and the GPR model is refined. This process continues until a predefined number of iterations is reached; at this point, the optimal parameters are used for the final CEEMDAN decomposition.

In this study, the previously extracted *U*_ce-p_ is decomposed using CEEMDAN with the optimized parameters. The decomposition performance is evaluated by the MSE between the reconstructed and original signals, and the results are presented in Table 1.

To identify the informative components and eliminate the noise, the Pearson Correlation Coefficient (PCC) is introduced to assess the linear correlation between each IMF and the original signal [38]. The PCC values range from −1 to 1, where values above 0.5 indicate a meaningful correlation, and it can be expressed as:

(7) $$p=\frac{\sum \left(X_{i}-\bar{X})(Y_{i}-\bar{Y}\right)}{\sqrt{\sum {\left(X_{i}-\bar{X}\right)}^{2}}\times \sqrt{\sum {\left(Y_{i}-\bar{Y}\right)}^{2}}}$$

**Figure 5:**
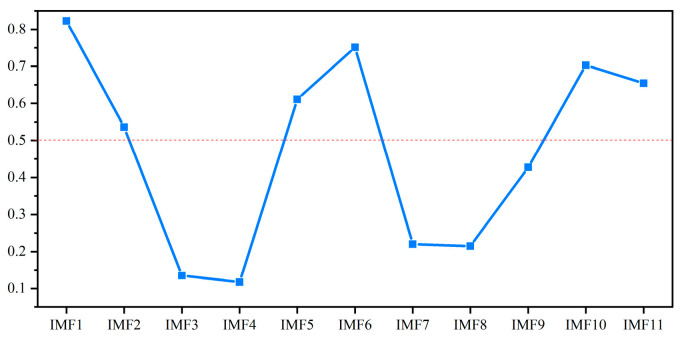
The PPC between each preliminary IMF component and the original signal. The red line is the selection threshold 0.5.

**Figure 6:**
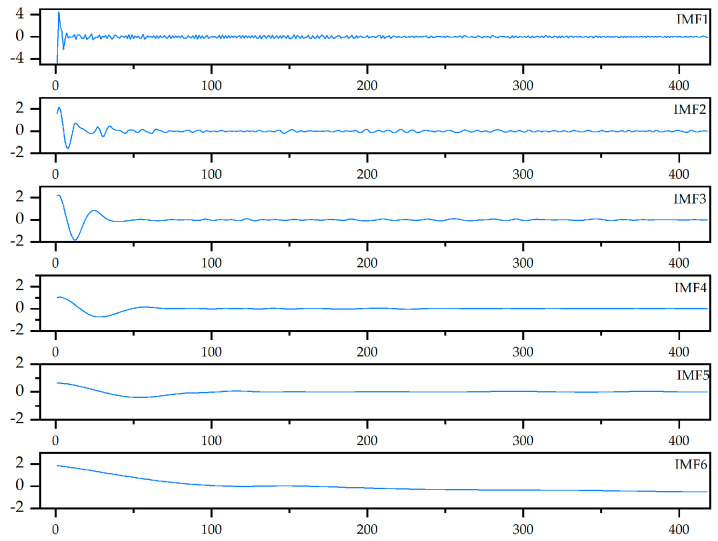
The selected optimal IMF.

Accordingly, 0.5 is the threshold for selecting the relevant IMFs from the 11 extracted components [39]. As shown in Figure 5, the six IMFs exhibit strong correlation, suggesting that they capture dominant signal features. The remaining IMFs likely contain noise or low-frequency drift. These six IMF components are selected to construct the fault feature vector for subsequent model training and prediction, as illustrated in Figure 6.

## 3. Performer-KAN-Based IGBT Failure Prediction

### 3.1. Performer-KAN Model

The Transformer has demonstrated a remarkable performance across various domains. However, its standard self-attention mechanism suffers from computational inefficiency. The time and space complexities of the original attention mechanism are *O*(*L^2^d*) and *O*(*L^2^ + Ld*), respectively, where *L* is the input sequence length and *d* is the feature dimension. As *L* increases, the quadratic growth in computational cost limits the model’s scalability in long-sequence scenarios. In the standard attention mechanism, the attention matrix *A* is computed by multiplying the query matrix *Q* and key matrix *K*, followed by the Softmax function. However, the nonlinearity of Softmax prevents the direct decomposition of *A* back into *Q* and *K*. Even applying nonlinear mappings to *Q* and *K* does not alleviate the complexity.

Krzysztof et al. proposed the Performer architecture to overcome this limitation, incorporating an efficient attention mechanism known as FAVOR+ (Fast Attention Via positive Orthogonal Random features) [40]. FAVOR+ provides an unbiased estimation of dot-product similarity by applying orthogonal random feature mappings, avoiding the explicit construction of the full attention matrix.

By mapping *Q* and *K* to the lower-dimensional representations $Q^{\prime }$ and $K^{\prime }$, FAVOR+ encodes similarity metrics and reduces attention computation’s time and space complexity to linear. As a result, Performer offers a scalable and theoretically sound solution for handling long sequences without relying on approximations. The mapped representations $Q^{\prime }$ and $K^{\prime }$ as well as the computation of the approximated attention score $A_{F}$, are formulated as follows:

(8) $$\phi (x)=\frac{s(x)}{\sqrt{M}}(f_{1}(W_{1}^{T}x),\;\ldots ,\;f_{1}(W_{M}^{T}x),\;\ldots ,\;f_{l}(W_{1}^{T}x),\;\ldots ,\;f_{l}(W_{M}^{T}x))$$

(9) $$Q^{\prime }=\phi (Q),\;K^{\prime }=\phi (K)$$

(10) $$A_{F}(Q,K,V)={\hat{D}}^{-1}(Q^{\prime }({\left(K^{\prime }\right)}^{T}V))$$

(11) $$\hat{D}=diag(Q^{\prime }({\left(K^{\prime }\right)}^{T}1_{L}))$$

**Figure 7:**
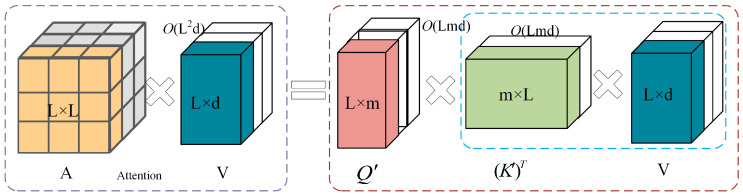
The random feature mapping approximation process for the attention matrix A.

Here, ϕ(x) is a mapping function, and it is realized via a random feature projection using an exponential kernel. *s*(*x*) is a normalization function to control the mapping scale. $f_{1},\;\ldots ,\;f_{l}$ is a set of functions that act on randomly mapped points $W_{i}^{T}x$, used to calculate the projection. *W* is a random matrix sampled from a Gaussian distribution. *M* is the dimension of the random feature mapping, $Q^{\prime },K^{\prime }\in R^{L\times r}$. The uniqueness of this mechanism lies in its reduced computational cost: The space complexity is *O*(*Lr + Ld + rd*) and the time complexity is *O*(*Lrd*), in contrast to the conventional attention mechanism with space and time complexities, respectively. When the sequence length *L* is large, Performer demonstrates significantly superior efficiency to standard attention. The approximation of the attention matrix A through random feature mapping is illustrated in Figure 7. Dashed blocks indicate the computation order and corresponding complexity at each stage.

The Kolmogorov–Arnold Network (KAN) is a novel neural network architecture inspired by the Kolmogorov–Arnold representation theorem. This theorem states that any multivariate continuous function can be represented as a finite superposition of univariate continuous functions. Based on this theorem, KAN models complex mappings by decomposing them into a set of learnable univariate functions, thereby offering a flexible and interpretable network structure [41].

Unlike conventional neural networks that apply fixed activation functions at each neuron, KAN places trainable spline-based activation functions on the edges (i.e., the weights), allowing the nonlinear transformation to be directly parameterized and optimized during training. In particular, KAN employs B-spline functions as activation functions. These are piecewise polynomial functions defined by control points and knots: The control points determine the shape of the curve, while the knots represent the segment boundaries of the spline. The activation function in KAN can be expressed as:

(12) ϕ(x)=w(b(x)+spline(x))

where *w* is a trainable weight and *b*(*x*) is the spline basis function. In this work, Sigmoid Linear Unit (SiLU) is chosen as the basis function, due to its smoother and non-monotonic characteristics compared to ReLU. This enhances the expressiveness of the network’s nonlinearity, making it particularly suitable for modeling complex functional relationships in KAN. The SiLU function is defined as:

(13) $$b(x)=\mathrm{SiLU}(x)=\frac{x}{1+e^{-x}}$$

**Figure 8:**
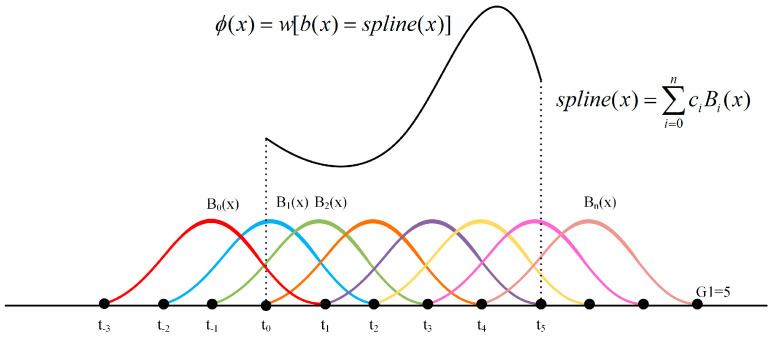
The learning process of a single nonlinear unit function.

Figure 8 depicts the learning process of a single nonlinear unit function *j*(*x*) to further illustrate the construction of spline-based activation in the KAN layer. First, a hyperparameter *G*_1_ is defined to specify the number of grid points used to partition the input space into nonlinear segments. In the illustrative example, *G*_1_ = 5, resulting in 5 spline sub-domains. Next, *n* B-spline basis functions are initialized, each associated with a learnable coefficient *c_i_*. The shape of each basis function is determined by its corresponding weight, and the weighted sum of all *n* splines yields an intermediate nonlinear mapping spline(*x*).

A smoothed nonlinear function, specifically the *SiLU*(*x*), is applied to the spline(*x*) to improve the smoothness and mitigate abrupt transitions, forming a composite curve. This result is multiplied by a learnable weight *w*, generating the final unit function *j*(*x*).

It is important to note that this process describes the learning of a single nonlinear unit. In prediction scenarios, the decoder output is projected onto multiple such units, each individually trainable. The final prediction is obtained by aggregating the outputs of all learned unit functions, ensuring flexible and expressive nonlinear modeling of the target signal.

**Figure 9:**
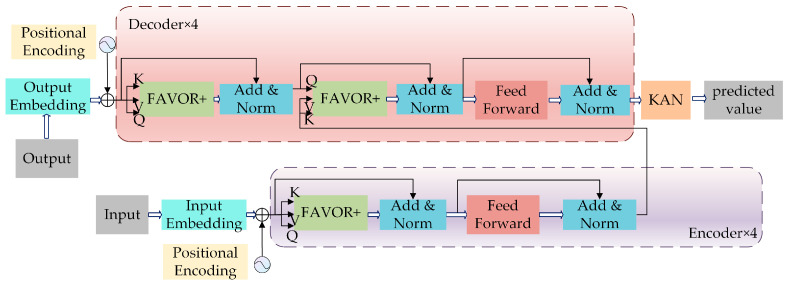
Performer-KAN model architecture diagram.

Since the multilayer perceptron (MLP) component in the Performer architecture is structurally complex and lacks an explicit mathematical formulation, it poses challenges for interpretability and functional analysis. To address this issue, this study replaces the MLP with the KAN to optimize the model further. The resulting Performer-KAN architecture is illustrated in Figure 9 and consists of an input embedding layer, positional encoding, an encoder, a decoder, and a KAN layer.

The embedding layer projects the input IMF sequences into high-dimensional feature vectors, which are then combined with positional encoding to incorporate temporal information. The encoder performs global modeling of these embedded features, capturing dependencies across different time steps. Each encoder layer comprises the FAVOR+ attention mechanism, a feedforward network (FFN), residual connections, and layer normalization. The FAVOR+ mechanism estimates the attention matrix $A_{F}$, while the FFN applies nonlinear transformations to enrich feature representations, computed as:

(14) Hffn=ReLu(AFW1+b1)W2+b2

(15) ReLU(x)=max(0, x)

where *W* is the linear layer weight, *b* is the bias vector. *H*_input_ is the features input to the current module, and *H*_processed_ is the features processed in the current layer. Residual connections and layer normalization together ensure a stable gradient flow and help prevent information degradation in deeper layers, computed as:

(16) $$H_{\mathrm{out}}=\mathrm{LayerNorm}(H_{\mathrm{input}}+H_{\mathrm{processed}})$$

(17) $$\mathrm{LayerNorm}(x)=\frac{x-\mu }{\sqrt{\sigma^{2}+\epsilon }}$$

The decoder mirrors the encoder’s structure, consisting of stacked sublayers. While the encoder encodes historical sequence patterns, the decoder generates the predicted target sequence. The hidden state corresponding to the final time step (*H_T_*) from the encoder output is extracted and passed into the KAN layer. The KAN begins by capturing the linear relationships within the input features through a basic linear transformation, computed as:

(18) $$\mathrm{Base}\;\mathrm{output}=W_{\mathrm{base}}H_{T}+b_{\mathrm{base}}$$

where $W_{base}\in R^{D\times C}$ is the weight matrix, $b_{base}\in R^{C}$ is the offset of the linear mapping, and *C* is the feature dimension of the output. Then, after the B-spline basis function mapping, the basis function and weights are computed:

(19) $$\left\{\begin{array}{l} \phi (H_{T})=\sum_{k}c_{k}\phi_{k}(H_{T})\\ \mathrm{Spline}\;\mathrm{output}=W_{spline}\phi (H_{T}) \end{array}\right.$$

where $\phi_{k}(H_{T})$ is basis functions and *W_spline_*$\in R^{C\times M}$ is the weight matrix. The range of *K* is [1, M]. The nonlinear mapping of high-dimensional data is achieved by combining multiple basis functions through a weighted summation using coefficients *C_k_*, which enhances the model’s feature representation capability. Here, *M* denotes the number of basis functions, *grid_size* determined by the number of spline sub-networks, and *Spline order* determined by the order of the basis functions. *M* can be expressed as:

(20) M=grid_size+Spline order

The final output is the prediction result, which is compared with the ground truth to evaluate the prediction accuracy.

(21) $$\hat{y}=\mathrm{Base}\;\mathrm{output}+\mathrm{Spline}\;\mathrm{output}$$

The pseudo-code for the Performer-KAN model is shown in Algorithm 1.
**Algorithm 1**: The learning algorithm of Performer-KAN **Input**: Time-domain features of fault parameters IMF    **Output**: Predicted IGBT fault parameter values    while not converge **do**1.         for epoch **do**2.               Embed IMF into a high-dimensional space with positional encoding.3.               Input IMF into Performer, compute random feature matrices *Q′*, *K′*, *V′*.4.               Use FAVOR+ mechanism to estimate attention weights:5.                       Attention_F_ (approximated via kernel feature mapping).6.               Apply residual connection and layer normalization to get *H_T_*.7.               Output of KAN is mapped to prediction dimension:8.                       $\hat{y}$ (spline basis mapping and weighted output).9.               Compute loss and backpropagate.10.        **end for**    **end while**

### 3.2. Performer-KAN -Based Failure Prediction Experiment

The fault prediction experiments based on the Performer-KAN model were developed and trained using Python 3.9.7 and the PyTorch-GPU 2.1.0 deep learning framework. The network architecture and parameter settings of the model are summarized in Table 2. The loss function is chosen as the MSE between the predicted and real values. Three metrics are employed to evaluate the predictive performance of the model: the coefficient of determination (R^2^), Root Mean Square Error (RMSE), and Mean Absolute Error (MAE). The R^2^ values closer to 1 represent a better model performance. Lower RMSE and MAE values indicate higher prediction accuracy. The corresponding formulas are shown in Equations (22)–(24).

(22) $$R^{2}=1-\frac{\sum_{i=1}^{N}{\left(y_{i}-{\hat{y}}_{i}\right)}^{2}}{\sum_{i=1}^{N}{\left(y_{i}-{\bar{y}}_{i}\right)}^{2}}$$

(23) $$RMSE=\sqrt{\left(\sum_{i=1}^{N}{\left|y_{i}-{\hat{y}}_{i}\right|}^{2}\right)/N}$$

(24) $$MAE=\left(\sum_{i=1}^{N}\left|y_{i}-{\hat{y}}_{i}\right|\right)/N$$

The Adam optimizer is employed, with an initial learning rate set to *Lr* = 1 × 10^−3^, a dropout rate of 0.2, a batch size of 32, and 300 training epochs.

To verify the computational efficiency of the Performer model, three metrics are employed to evaluate training cost: training time per epoch (minutes), GPU utilization (%), and training time per batch (seconds). The evaluation begins by initializing monitoring variables, including GPU utilization and timing statistics for each batch and epoch. A dedicated function is defined to retrieve the GPU usage information during training continuously. The training loop is integrated with GPU monitoring and time measurement routines. Specifically, GPU utilization is recorded in real-time, and torch. cuda. Event is used to measure training durations. Batch-wise and epoch-wise timings are independently captured. Upon completion of training, the average GPU utilization, batch training time, and epoch training time are calculated.

## 4. Experimental Results and Analysis

Experiments were conducted on the extracted fault parameter sequences to validate the applicability and effectiveness of the proposed model. The dataset was divided into a training set (80%) and a testing set (20%). In addition, several classical time-series prediction models and Transformer-based variants were introduced for comparison. This allows for a comprehensive evaluation of the Performer-KAN model in terms of both the prediction accuracy and computational efficiency.

### 4.1. Analysis of IGBT Fault Prediction Results Based on Performer-KAN Modeling

To validate the applicability and effectiveness of the CEEMDAN method, this study employs three types of input sequences: the original *U*_ce-p_, the features processed by Kernel Principal Component Analysis (KPCA), and the IMFs obtained through CEEMDAN decomposition, to train and test the Performer-KAN model. The corresponding results are presented in Table 3.

**Figure 10:**
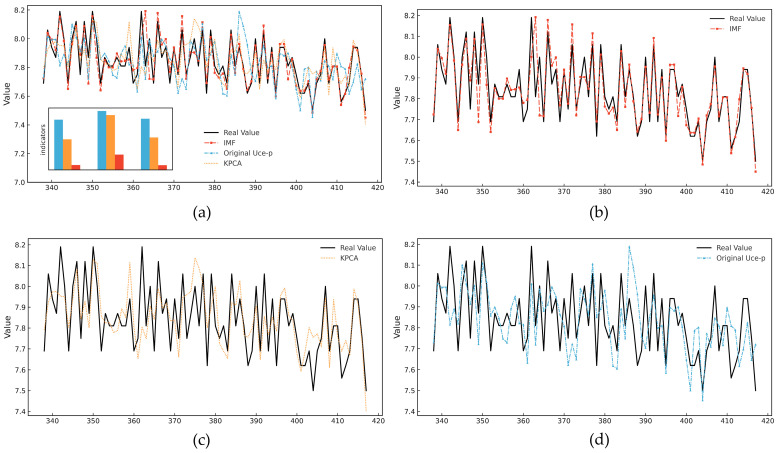
Results of Performer-KAN’s comparison of the predicted values with real values for different input sequences: (**a**) is the overall comparison figure, including the error comparison. (**b**–**d**) are the comparison results when the input sequences are IMF, KPCA, and the original *U*_ce-p_.

Experimental results show that the model performs best across all evaluation metrics when using IMF sequences as input. Compared with the original *U*_ce-p_ data and KPCA, the IMF-based model achieves an R^2^ exceeding 0.98 and significantly reduced MAE and RMSE, demonstrating its superior prediction accuracy. These results indicate that the CEEMDAN method effectively separates key signal features from noise, significantly improving both the robustness and accuracy of the fault prediction model. Figure 10 compares the predicted values and the ground truth for the three types of input sequences. It is observed that when using the IMF sequences, the predicted curves closely match the actual values in both the trend and amplitude. In contrast, when using the original *U*_ce-p_ data, the model captures only the general trend due to redundant information, rleading to prediction amplitude deviation. Although KPCA enhances feature extraction to some extent, it still struggles to accurately capture local high-frequency variations, leading to noticeable fitting errors.

Therefore, in subsequent comparative experiments, the IMF sequences are consistently selected as the fault feature input to further evaluate the proposed method’s comprehensive performance.

### 4.2. Comparative Experimental Results Analysis

This study compares the Performer-KAN model against six representative deep learning models, including RNN, LSTM, and Temporal Convolutional Network (TCN), to validate its effectiveness. These reflect three major technical paradigms in time series modeling: recurrent iteration, gated mechanisms, and convolutional expansion. All three have shown solid performance in time-series forecasting tasks. In addition, two Transformer variants are introduced for comparison: the Transformer-KAN model, which replaces the original MLP with KAN, and the Informer model, another improved Transformer architecture. These models are selected to comprehensively evaluate the proposed method’s advantages over classical and Transformer-based alternatives. All baseline models were equivalent.

The experimental results in Table 4 demonstrate that the Performer-KAN model performs best across all evaluation metrics, with an R^2^ of 0.9841, MAE of 0.049, and RMSE of 0.0153. These results highlight its superior fitting capability and robustness in time-series modeling. Compared to the original Performer model, Performer-KAN significantly improves prediction accuracy, suggesting that incorporating KAN enhances the model’s ability to capture complex fault characteristics and improves generalization.

Relative to conventional time-series models such as LSTM, RNN, and TCN, Performer-KAN exhibits a stronger performance in modeling long-term dependencies and capturing degradation trends, thereby reducing the prediction lag and amplitude deviation. Although the Transformer-KAN model achieves a slightly lower MAE, it performs slightly worse in other metrics, indicating that the Performer-KAN model offers a better overall performance. Informer, while moderately effective in specific tasks, suffers from amplitude amplification during long sequence modeling, compromising its stability and prediction accuracy.

**Figure 11:**
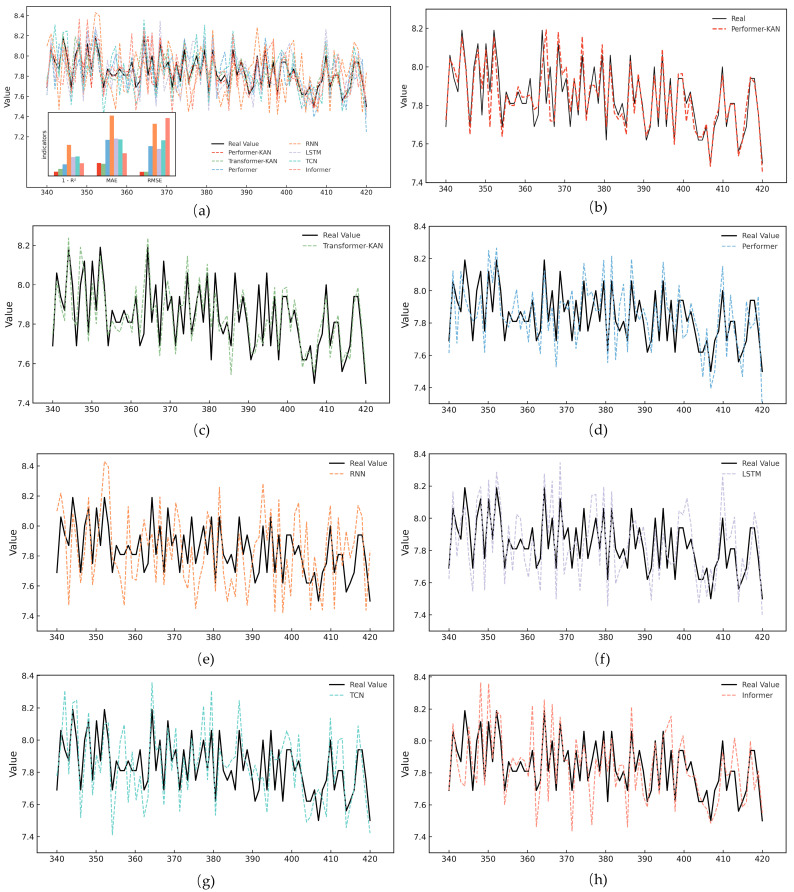
Results of the different models’ comparison of predicted values with real values: (**a**) is the overall comparison figure, including the error comparison, (**b**–**h**) are the results of comparing the real values with the predicted values of the models, respectively.

To further illustrate the model performance, Figure 11 visualizes the predicted and real sequences across models using IMF inputs. The Performer-KAN model matches the ground truth in trend and magnitude, reinforcing its superiority and reliability in IGBT fault prediction tasks.

**Figure 12:**
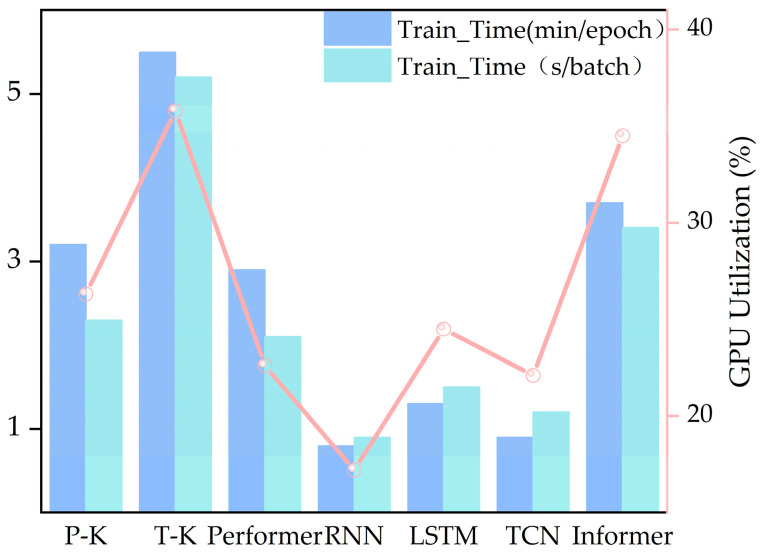
The comparative results of the computational efficiency of the models.

To evaluate the computational efficiency, three metrics were recorded for each model using IMF sequences: training time per epoch (min/epoch), training time per batch (s/batch), and GPU utilization, as shown in Figure 12. Among all the models, the Transformer-KAN incurs the highest computational overhead, reflecting an increased resource consumption despite an improved modeling capacity.

In contrast, the Performer-KAN achieves notable efficiency improvements, requiring only 3.2 min per epoch, 2.3 s per batch, and utilizing 26.3% of the GPU on average. This is primarily attributed to the FAVOR+ mechanism, which significantly reduces the time and memory complexity, enabling a high-accuracy prediction with lower latency and hardware demands.

The TCN benefits from parallel convolutional operations among traditional models, leading to the fastest training speed. The RNN and LSTM are slower due to sequential dependencies, with RNN being slightly quicker than LSTM due to its simpler architecture.

For Transformer-based variants, Informer’s training efficiency lies between the Transformer-KAN and the Performer-KAN. Although it adopts a sparse attention mechanism, its acceleration is less effective than FAVOR+ in this experimental setting.

The Performer-KAN demonstrates the best balance between prediction accuracy, training efficiency, and computational resource utilization. Its scalable performance and low complexity make it well-suited for practical engineering applications. While Transformer-KAN shows marginal improvements in MAE, its high computational cost may limit the deployment in real-time environments. Though fast and resource-efficient, traditional models like the RNN, LSTM, and TCN fall short in delivering high-precision degradation predictions.

## 5. Conclusions

This study proposes a novel IGBT fault prediction method based on a hybrid Performer-KAN architecture. By integrating the FAVOR+ and KAN, the model effectively balances prediction accuracy with computational efficiency. The CEEMDAN decomposition technique, optimized via Bayesian inference, is also introduced for fault feature preprocessing. Extensive experiments demonstrate that the Performer-KAN model outperforms several benchmark models across all evaluation metrics. It achieves the highest R^2^ and the lowest MAE and RMSE, confirming its superior predictive accuracy and generalization capability. Furthermore, the Performer-KAN framework achieves substantial improvements in training efficiency while minimizing the computational overhead, making it highly suitable for real-time industrial applications.

Despite its promising performance, the proposed framework has several limitations. First, the current experiments focus on single-parameter degradation signals. Future work will consider multivariate feature fusion to capture more complex fault characteristics.

## Figures and Tables

**Table 1:** Bayesian optimization results.

Noise Increment	Number of Integrations	MSE	Number of IMF
0.347	32	0.0091	11

**Table 2:** The network architecture and parameter settings of the model.

Parameters	Layer Name	Output Dim
d_model = 128	Encoder Input	(318, 6)
*N* = 4	Input Embedding	(318, 128)
kernel_type:exp	Encoder Block × 4	(318, 128)
*grid_size* = 5	Decoder Input	(80, 1)
*spline_order* = 7	Decoder Embedding	(80, 128)
	Decoder Block × 4	(80, 128)
	KAN	(80, 1)

**Table 3:** Prediction results of Performer-KAN with different input sequences.

Input	R^2^	MAE	RMSE
Original Uce-p	0.8398	0.1880	0.1631
KPCA	0.9025	0.1753	0.1033
IMF	0.9841	0.049	0.0153

**Table 4:** Prediction results of the different models.

Model	R^2^	MAE	RMSE
Performer-KAN	0.9841	0.049	0.0153
Transformer-KAN	0.9732	0.046	0.0158
Performer	0.956	0.137	0.113
RNN	0.8822	0.229	0.198
LSTM	0.928	0.142	0.103
TCN	0.926	0.139	0.135
Informer	0.9519	0.086	0.22

## Data Availability

The data are published by NASA. Website: https://data.nasa.gov/dataset/. Accessed on 10 January 2023.
